# Fibrous Tumor of the Inferior Maxilla

**Published:** 1845-05

**Authors:** 


*Fibrous Tumour of the Inferior Maxilla.*—Mr. Hodgson then presented another tumour, which he had removed this morning from the inner portion of the inferior maxilla of a woman; it was easily removed. It was composed of a hard fibrous substance, interspersed with numerous spiculæ of bone.

Mr. Hodgson said that he removed a similar disease from a woman some years since; the disease returned in a very sluggish manner in the condyle of the maxilla, which he had not thought necessary to disarticulate at the time of taking out the diseased portion, but had cut the bone through just below it. He regretted now that he had not disarticulated the condyle, for he left only a small portion of the bone behind. The case, however, was going on very well. Mr. Hodgson does not believe that this disease is malignant, as some cases had been cured by the application of iodine and mercury, and they generally got well if completely removed.

